# Examining Public-Facing Hospice Medical Aid in Dying Participation Policies in Legalizing US Jurisdictions

**DOI:** 10.1093/geroni/igaf122.868

**Published:** 2025-12-31

**Authors:** Kira Philips, Sanjana Kumar, Todd Becker, Elizabeth Luth, Veda Kota, Samuel Nemeth, Elissa Kozlov, Paul Duberstein

**Affiliations:** Brown University, Providence, Rhode Island, United States; Brandeis University, Waltham, Massachusetts, United States; WashU Medicine, St. Louis, Missouri, United States; Rutgers University, New Brunswick, New Jersey, United States; Rutgers School of Public Health, Piscataway, New Jersey, United States; Purdue University, West Lafayette, Indiana, United States; Rutgers School of Public Health, Piscataway, New Jersey, United States; Rutgers University, New Brunswick, New Jersey, United States

## Abstract

Medical aid in dying (MAID) permits qualified individuals to self-administer a medication to hasten their death. Although currently legal for roughly 74 million Americans across 10 states and Washington, DC, little is known about hospice organizational policies toward participation. This study aimed to characterize the availability and content of public-facing hospice MAID participation policies across legalizing jurisdictions. We used 2024 Centers for Medicare & Medicaid Services’ Hospice General Information data to identify all Medicare-certified hospices in legalizing jurisdictions. Random selection of 15.9% of California hospices, due to increased representation, and inclusion of all hospices from the remaining jurisdictions resulted in a sample of 724 hospices. We identified public-facing policy availability by searching sample hospices’ websites. Policy content was independently extracted by two analysts using a researcher-constructed tool and subsequently coded as “yes,” “no,” or “unreported.” We described policy availability and content via frequency and percentage. Results indicated that only 5.4% of hospice websites contained a public-facing MAID participation policy. Policy content across reporting hospices was highly variable. Whereas 69.2% reported on the provision of patient about local MAID laws, only 20.6% indicated having a protocol outlining patient MAID initiation. When reported, language was often vague, complicating discernment of how hospices formatively participated in MAID. For instance, 46.2% did not report whether or not employed providers could prescribe MAID medications. The lack of availability and specificity in these policies likely affects patients’ informed decision making about hospice selection, which may then affect receipt of goal-concordant care. Greater organizational transparency is needed.

